# Outcomes and Impact of Fragility Fracture among Geriatrics Patients who Underwent Hip Surgery in Hospital Kuala Lumpur

**DOI:** 10.5704/MOJ.2203.012

**Published:** 2022-03

**Authors:** HT Lim, EGM Chong, WK Yau, H Abdul-Halim

**Affiliations:** Geriatric Unit, Department of Internal Medicine, Hospital Kuala Lumpur, Kuala Lumpur, Malaysia

**Keywords:** fragility fracture, hip surgery, orthogeriatric, functional outcome, economic burden

## Abstract

**Introduction:**

Fragility fractures are common in the elderly. It is associated with increased mortality, reduced mobility, and poorer quality of life. In addition, post-operative functional outcomes are limited locally.

**Materials and methods:**

A cross-sectional phone interview was conducted with elderly patients who underwent hip surgery or their caregivers between March 2019 and Feb 2020, at least six months after the operation.

**Results:**

A total of 137 cases were approached, and 77 subjects completed the interview (58.4%), among which 54/77 (70.1%) were female, and 66/77 (85.7%) were caregivers. The proportion of subjects who could ambulate independently dropped from 66/77, prior to fracture, to 17/77 post-surgery. We noted a significant deterioration in the modified Barthel Index from the median of 100 (IQR = 0) to 91 (IQR 25.5; p <0.001). There was also a significant decline in the self-perceived physical strength of 30% (IQR 30, p <0.001); and in the functionality of 35% (IQR40; p <0.001). A total of 48/77 (62.3%) returned to their original residence, while 5 cases (6.5%) were institutionalised, and 14/77 (18.2%) died prior to the survey. Thirty-six subjects reported additional costs in the care of patients, ranging from RM100 to RM6000 (USD25 to USD1450) per month.

**Conclusion:**

Decline in physical and functional status is closely related to the quality of life as the majority reported a poorer health status after the fracture. Although this study is limited by the small sample size, it provided insights into patients' experiences and household burdens. Hence, well-coordinated services and monitoring are important for better outcomes.

## Introduction

A fragility fracture is sustained from low-energy trauma, such as a fall from a standing height or less^1^. One in three women and one in five men over the age of 50 will experience osteoporotic fractures^1,2^. The incidence of osteoporosis and fragility fracture increases with age. With the increase in life expectancy and multiple co-morbidities, fragility fracture confers a significant impact on health and long-term outcomes in the elderly population. The Asian Federation of Osteoporosis Societies projected a 3-fold increase in hip fractures, and Malaysia is no exception^3^. Therefore, reviewing the care pathway in fracture management and incorporating new models of care are urgently needed^4^.

Numerous studies show the benefits of collaboration between orthopaedic and geriatric teams in managing elderly patients who sustained a hip fracture. It optimises early surgical intervention, reduces perioperative complications, and reduces morbidity and mortality rates^5,6^. Key elements of good care encompass prompt admission and comprehensive assessment, minimal delay to surgery, accurate and well-performed surgery, early rehabilitation, and secondary prevention^7^.

With an average mortality rate of around 17.7% among this patient population, factors influencing their health after fracture are important^8^. The quality indicators that improve the outcome include secondary fracture prevention, systematic pain assessment and malnutrition, and pressure sore prevention^9^. On the other hand, frailty, sarcopenia, co-morbidities, high American Society of Anaesthesiologists (ASA) grades, delay in operation, and poor socioeconomic factors are associated with poorer long-term outcomes^10^. Frequently, demands in the level of care and costs in managing patients are proportionate to the degree of decline in patients’ functional status. However, local data on functional outcomes post-surgery are lacking. Hence, this study aimed to study the clinical and functional outcomes of geriatric patients who had hip surgery.

## Materials and Methods

This is a cross-sectional study on all the geriatric patients, who were more than 60 years old, who underwent hip surgery in Hospital Kuala Lumpur (HKL) after the establishment of the orthogeriatric liaison services in March 2019. We obtained ethical approval from the Medical Research and Ethical Committee (MREC), of the Malaysian Ministry of Health. Patients referred to the geriatric team from orthopaedic wards during the perioperative period were recruited. Operation theatre lists were reviewed to maximise the case detection. Cases from March 2019 till Feb 2020 were then short-listed and approached. Patients or next-of-kin who declined to participate and non-responders were excluded. Verbal consent was obtained prior to the phone survey. For those who consented, a trained medical officer performed the phone survey using a pre-set questionnaire at least six months after discharge from the hospital. The phone survey took around 15 minutes to complete, and it was tested in a pilot trial earlier. Cases who were uncontactable after two attempts on different occasions were considered as non-responders. We performed the survey from May until August 2020.

Data collection included mobility status, functional status, type of walking aid, location of residence, additional cost of care, post-discharge complications, and mortality. We used the modified Barthel index to assess patients' functional status. Self-perceived health status was measured on physical strength and performance with a self-rated scale of 0 to 100. All reported additional costs were recorded in Malaysian Ringgit (RM).

Regarding data analysis, SPSS version 25 was used for descriptive data analysis. We used the Wilcoxon test to analyse non-parametric data sets. Statistical significance was set at the p-value of <0.05.

## Results

**Fig. 1:**
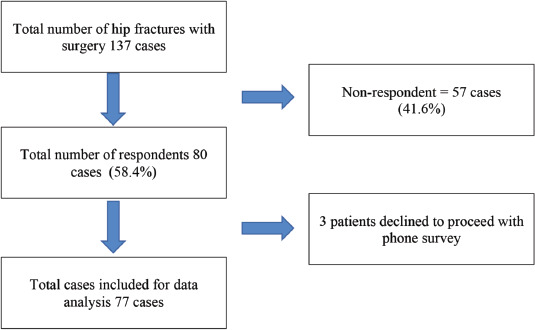
The recruitment flow and number of respondents.

A total of 137 cases were short-listed, and 80 patients responded (58.4%). The recruitment flow and the number of respondents are summarised in Fig. 1. The majority of respondents were caregivers, comprising 68 cases (85%). Three cases declined to participate. Patient characteristics are summarised in Table I. The median duration from operation to the phone interview was 46 weeks (IQR 34-63).

**Table I:** Summary of patient characteristics

		Number	Percentage, %
Age	Mean age 77.4 (Standard deviation 9.1)		
	<75 years old	33	42.9
	≥75 years old	44	57.1
Gender	Male	23	29.9
	Female	54	70.1
Median duration from admission to operation (IQR), days Ethnicity	6 (4-12)		
	Malay	25	31.3
	Chinese	35	43.8
	Indian	18	22.5
	Others	2	2.5
Pre-operation ASA Classification	1	7	9.1
	2	38	49.4
	3	8	10.4
	Missing	24	31.2
Types of fracture	Intracapsular	36	46.8
	Intertrochanteric	36	46.8
	Subtrochanteric	4	5.2
	Missing	1	1.3
Types of surgery	DHS/PFN	43	55.8
	Hemiarthroplasty	18	23.4
	Total hip replacement	15	19.5
	Missing	1	1.3

Abbreviations: DHS = dynamic hip screw, PFN = proximal femur nail, ASA Classification = American Society of Anaesthesiologists Classification

**Fig. 2:**
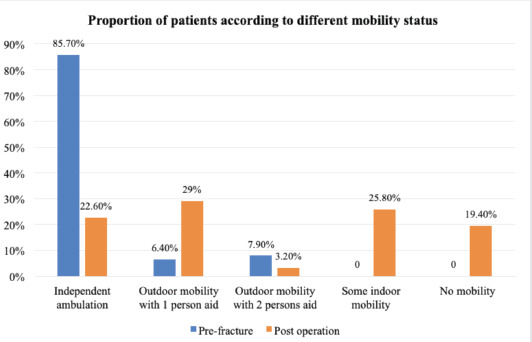
Comparison of patients’ mobility status between pre-fracture and post-operative periods.

A total of 66/77 (85.7%) of patients could ambulate independently without aid prior to the index fracture. However, only 17/77 (22.6%) were able to do so six months after the operation (Fig 2). 41/77 (53.2%) of patients achieved mobility with walking aids (quadripod or walking frame), while 12/77 (15.6%) of patients required a wheelchair for ambulation. Of note, only 29/77 reported receiving some rehabilitation after discharge. The intensity and frequency of physiotherapy were not quantified.

On the other hand, most individuals had good functional status prior to the fracture. The median Modified Barthel Index (MBI) declined from 100 to 91 (IQR 25-100; p <0.001) (Table II). 61.1% of the subjects perceived that their health status had deteriorated. When the subjects were asked about their self-perceived physical strength and functionality, with a baseline reference of 100, the median reductions from baseline were 30 and 40, respectively. 52/77 (67.5%) reported having a poorer health status after the fracture. A total of 30/77 (38.9%) required assistance in more than one activity of daily living (ADL). The median number of assisted ADLs was 2 (IQR 0-7). A total of 48/77 (62.3%) of cases reported that they had returned to their original residence, while 5 cases (6.5%) were institutionalised, 12/77 (15.6%) of cases moved and lived with other family members.

**Table II:** Comparison of outcome measures before and after hip fracture (minimum after six months)

Outcome measures	Prior to fracture Median (IQR)	At the point of the interview Median (IQR)	p-value
Modified Barthel Index	100 (100–100)	91.5 (72.5–98)	<0.001
Self-perceived physical strength	100 (default value)	70 (50 to 80)	<0.001
Self-perceived functionality	100 (default value)	60 (40 to 80)	<0.001

Abbreviations: IQR = interquartile range, ADL = activity of daily living

Fourteen cases (18.2%) died before our survey. Other reported complications are summarised in Table III. Among those admitted, three cases had direct complications related to the hip surgery, which included hip dislocation, implant failure with septic arthritis, and operation site infection.

**Table III:** Summary of reported complications after a hip surgery

Complication variables	No. of cases	Percentage
Mortality	14	18.2
Chronic pain	39	50.6
Subsequent admission (s)	15	19.4
Recurrent fracture	1	1.3
Pressure sore	4	5.2
Infection	6	7.8
Re-operation	3	3.9
Fall14	18.2	
Others	8	10.4

When estimating the household burden of patients who underwent hip surgery, we enquired about the cost of nursing care, domestic help, medication, food, and others. Thirty-one subjects reported additional costs in managing persons with hip fractures, ranging from RM100 to RM6000 (USD25 to USD1450) per month. On average, each household spent an additional RM711.40 per month managing one patient. Among the commonly reported expenditures, the cost for nursing care, an average of RM417 per month, and for domestic helpers, an average of RM130 per month were the major contributors. Diapers were the most frequently reported item purchased regularly.

## Discussion

More than 60% in our cohort reported a poorer health status during the survey. This was observed in other studies, in which significantly poorer quality of life was noted among subjects with hip fracture^14-16^. Poorer functional recovery is associated closely with poorer quality of life. Cornelis *et al* showed that pre-fracture frailty was negatively associated with health status, self-rated health, and physical capability17. In order to optimise quality of life, efforts should be focused on providing individualised care planning until optimal outcomes are achieved.

Generally, the excess mortality rate was observed in the first 6 to 12 months after the index fracture. Some studies showed that standardised mortality ratio would drop to baseline rate after about two years^18^. In our study, 71.4% of the mortality cases occurred within the first six months after a hip surgery. As demonstrated in other studies, factors such as male gender, high ASA score, major post-operative complications, ambulation with an assistive device, and household ambulation were associated with higher mortality rate^18^-^20^. We did not report on the causes for the mortality cases due to uncertainty from the family of the deceased and the limited accessibility of mortality datasets. Interestingly, other studies showed that household ambulation has the highest hazard ratio (HR = 2.19) and this could probably relate to either pre-existing frailty, sarcopenia, or exaggerated rate of physical deconditioning after the hip fracture^18-20^. In our cohort, 10 out of 14 mortality cases were older than 74 years old, suggesting that older patients are more vulnerable after an operation.

With regards to the economic burden of fragility fracture, there are numerous studies that estimated the direct costs of the fracture^21-23^. However, studies on indirect costs were rather limited. A systematic review reported that the cost of treatment for fragility hip fracture in Asia ranged between US$774 to US$14K (median US$2943)^24^. Several studies demonstrated that fragility fracture per se contributes to excessive costs on top of osteoporosis. It arises from the extra care required after hip fractures, such as physical therapy visits, home visits, nursing home stays, and hospital admissions^21,22^. Indirect costs such as productivity losses in the household should also be considered. This is valuable for services development and planning in the local region, as the care of elderly patients depends heavily on family members. Cost containment and coordination of post-operative care services are important for the economic and social system. This is the first study in Malaysia that attempted to evaluate the financial burden of a fragility fracture on households. With a median household income in Malaysia of RM5873 (US$1410) per month in 2019, the additional cost in caring for seniors estimated in our study can easily consume 12% or more of the household income^25^. Our method depended greatly on information volunteered by the respondents, and it might not capture other indirect costs, including efforts contributed by children and loss of potential income when the younger generation had to look after their seniors. In our local setting, caregiving depends significantly on family members as a community support system is still lacking, especially in tackling immobility and functional decline. When a household is overburdened, choices probably must be made between daily living expenses and quality of care for the fragility fracture survivors. This stresses the necessity of a supporting social system for vulnerable ones. Also, further studies are needed to explore the financial burden of fragility fracture in the long run.

This study has several limitations, with the small proportion of respondents in the survey. This may result in underestimating the mortality rate among patients who underwent hip interventions. In addition, as most respondents were caregivers, measurements requiring subjective answers may not fully reflect the patients' situation. This is especially true for the impact on psychological well-being. Formal assessment with tools such as the Geriatric Depression Scale (GDS) should be incorporated into clinical assessment during follow-up reviews. The study was also subject to recall bias as the median duration post-operation was ten months. The survey response rate also varied between different ethnicities. For instance, 66% of Chinese subjects responded to our survey compared to 53% of Malay subjects. This might affect the accuracy in estimation, especially in assessing the impact on the household financial burden.

## Conclusion

In the future, the findings from our study will serve as a foundation for more measures to be instituted either postoperatively or after hospital discharge. Population with fragility fracture requires more comprehensive discharge and rehabilitative planning. Carer training, closer monitoring, dietary intervention, tailored physical prescription and case coordination are among the aspects of care that can be improved locally. In conclusion, fragility hip fracture has a profound impact on patients’ mobility, functional status, and quality of life. The care for patients with hip surgery and the associated financial implication should be part of a multidisciplinary management.
